# Altered Autophagy-Associated Genes Expression in T Cells of Oral Lichen Planus Correlated with Clinical Features

**DOI:** 10.1155/2016/4867368

**Published:** 2016-02-15

**Authors:** Ya-Qin Tan, Jing Zhang, Ge-Fei Du, Rui Lu, Guan-Ying Chen, Gang Zhou

**Affiliations:** ^1^The State Key Laboratory Breeding Base of Basic Science of Stomatology (Hubei-MOST) and Key Laboratory of Oral Biomedicine Ministry of Education, School and Hospital of Stomatology, Wuhan University, Luoyu Road 237, Wuhan 430079, China; ^2^Department of Oral Medicine, School and Hospital of Stomatology, Wuhan University, Luoyu Road 237, Wuhan 430079, China

## Abstract

Oral lichen planus (OLP) is a T cell-mediated inflammatory autoimmune disease. Autophagy has emerged as a fundamental trafficking event in mediating T cell response, which plays crucial roles in innate and adaptive immunity. The present study mainly investigated the mRNA expression of autophagy-associated genes in peripheral blood T cells of OLP patients and evaluated correlations between their expression and the clinical features of OLP. Five differentially expressed autophagy-associated genes were identified by autophagy array. Quantitative real-time RT-PCR results confirmed that* IGF1* expression in the peripheral blood T cells of OLP patients was significantly higher than that in controls, especially in female and middle-aged (30–50 years old) OLP patients. In addition,* ATG9B* mRNA levels were significantly lower in nonerosive OLP patients. However, no significant differences were found in the expression of* HGS*,* ESR1*, and* SNCA* between OLP patients and controls. Taken together, dysregulation of T cell autophagy may be involved in immune response of OLP and may be correlated with clinical patterns.

## 1. Introduction

Oral lichen planus (OLP) is a common T cell-mediated chronic inflammatory disease with the characteristics of adult onset, female predilection, and autoimmune attack by infiltrating T cells in the oral mucosa and extraoral lesions on areas including the skin, genitalia, and nails [1, 2]. OLP affects 1-2% of the general adult population and is considered as a potentially malignant disorder with a malignant transformation rate of 0-1% according to the World Health Organization (WHO) [3]. Clinically, OLP mainly manifests three different forms: reticular, atrophic, and erosive [4]. They are generally simplified into two categories: erosive (erosive lesions) and nonerosive (reticular and atrophic lesions) [5]. Histologically, OLP is characterized by a dense infiltration of T cells in the lamina propria, basement membrane disruption, and the degeneration of basal keratinocytes [4, 6]. Extensive evidence has suggested that the T cell-mediated immune responses have pivotal roles in the onset and perpetuation of this disorder [4, 6–9]. The immunopathogenesis of OLP may involve antigen presentation, T cell activation and migration, and keratinocyte apoptosis [4]. In OLP, T cells are activated when presented with antigens by major histocompatibility complex (MHC) classes II and I molecules [3, 4]. Following antigen recognition, activated cytotoxic T cells may trigger keratinocyte apoptosis and release chemokines that attract additional helper T cells into the developing OLP lesion. The activated helper T cells may in turn activate cytotoxic T cells and participate in the antigen presentation and keratinocyte apoptosis [2, 3]. Our previous studies revealed that an imbalance between Th1/Th2 immune response was associated with disease onset and showed that OLP was featured by a Th1 cytokine bias and a Th1-biased pattern of upstream transcription factors expression. Thus, the Th1 immune response plays a dominant role in OLP [4, 6, 7].

As a strictly regulated lysosomal degradation pathway, autophagy is crucial for maintaining intracellular homeostasis and normal development [10]. Autophagy is involved in various innate and adaptive immune processes, including pathogen recognition and destruction, antigen processing for MHC presentation, lymphocyte development and function, and inflammatory regulation [11, 12]. After being stimulated by T cell receptor activation upon antigen recognition, autophagy can be induced in T cells and is required for T cell proliferation, differentiation, survival, and death [13, 14]. Autophagy has emerged as a fundamental trafficking event in mediating T cell response and regulating T cell immunity [13, 15]. Thus, T cell autophagy is hypothesized to be involved in the immunopathogenesis of OLP.

Defects in autophagy-associated genes and recruitment of autophagy-associated proteins are essential for autophagic dysfunction [16]. In recent years, the dysregulation of autophagy-associated genes has been recognized to increase the susceptibility to diverse diseases, including inflammation, autoimmune disorders, and cancer [17–19]. Barrett et al. reported that three autophagy-associated genes (*IRGM*,* NOD2*, and* ATG16L1*) were involved in autoinflammatory Crohn's disease [20]. Besides, elevated expression of* ATG5* in T cells may contribute to the T cell-mediated inflammatory demyelination in multiple sclerosis [21]. Furthermore, aberrant* ATG5*,* ATG7*,* LC3*,* HSPA/HSP70*,* UVRAG*, and* IRGM* expression levels have been implicated in the dysregulated T cell immune response of systemic lupus erythematosus [22]. These findings have suggested the immunological role of T cell autophagy in human diseases. However, it is not yet known whether autophagy in T cells plays a role in the pathogenesis of OLP. Therefore, reverse transcription polymerase chain reaction (RT-PCR) autophagy arrays were performed using RNA extracted from the peripheral blood T cells of OLP patients to screen 84 genes that encode components of the molecular machinery and key regulators of autophagy. Furthermore, differential gene expression between OLP patients and controls was validated using quantitative real-time RT-PCR methods. Finally, correlations between the mRNA expression of these autophagy-associated genes and the clinical features of OLP were analyzed.

## 2. Materials and Methods

### 2.1. Study Participants

Twenty-five patients with OLP and 13 age-sex-matched healthy controls were recruited from the Department of Oral Medicine, School and Hospital of Stomatology, Wuhan University. Informed consent was obtained from each subject before the study began. The Ethical Committee Board of the School and Hospital of Stomatology, Wuhan University, approved this study according to the Declaration of Helsinki on human subject protection. The group of OLP patients consisted of 13 females and 12 males with a mean age of 40.2 years and an age range of 19–57 (40.2 ± 2.4 years). There were 8 females and 5 males with an age range of 20–55 (34.5 ± 3.3 years) in the control group. The subjects were enrolled according to the criteria described in our previous studies [4]. Briefly, all study subjects did not suffer any other disorders or receive any treatment within recent 3 months. In addition, OLP can be subdivided into nonerosive form (NEOLP) and erosive form (EOLP). The clinical characteristics of the study subjects were listed in Table 1.

### 2.2. T Cells Isolation and Purification

Peripheral blood mononuclear cells (PBMCs) were collected from subjects by venipuncture and separated by density-gradient centrifugation over Ficoll-Hypaque solution (Tianjin Haoyang Biological Manufacture Co. Ltd., Tianjin, China). T cells were negatively selected from PBMCs using biotin human T lymphocyte enrichment cocktail and streptavidin particles (BD Biosciences, San Jose, CA, USA) together with an IMag*™* cell separation system (BD Biosciences).

### 2.3. RNA Extraction

Total RNA was extracted from 1 × 10^7^ T cells using an RNeasy® mini kit (QIAGEN GmbH, Hilden, Germany). Potential genomic DNA contamination was removed from the samples by treatment with RNase-free DNase (QIAGEN) for 15 min at room temperature. Concentration and purity were determined using a NanoDrop 1000*™* spectrophotometer (Thermo Fisher, Dubuque, IA, USA). The quality of the isolated RNA samples was confirmed by examining the integrity of 28S and 18S ribosomal RNA bands through electrophoresis on agarose gels containing formaldehyde.

### 2.4. Autophagy Array Assay

The human RT^2^ profiler PCR autophagy array (QIAGEN) was used to study the expression of 84 autophagy-associated genes in the peripheral blood T cells of OLP patients and controls. Briefly, using an RT^2^ first Strand kit (QIAGEN), 1 *μ*g total RNA obtained from T cells was incubated with the kit's genomic DNA elimination mixture at 42°C for 5 min and then transferred to ice for no less than 1 min to remove any residual DNA contamination. The kit's reverse transcription mixture was added to the purified RNA sample. The mixture was incubated at 42°C for 15 min and then 95°C for 5 min to convert total RNA back into cDNA. After cDNA synthesis, real-time RT-PCR was performed using RT^2^ SYBR® Green master mix (QIAGEN), according to the manufacturer's instructions. The amplification data (fold changes in the threshold cycle [Ct] values of all the genes) were analyzed by the ΔΔCt method.

### 2.5. Quantitative Real-Time RT-PCR Confirmation

Next, we inspected 5 autophagy-associated genes that were identified to be differentially expressed between OLP patients and controls by the autophagy array: insulin-like growth factor 1 (*IGF1*), autophagy related 9 homolog B (*ATG9B*), hepatocyte growth factor-regulated tyrosine kinase substrate (*HGS*), estrogen receptor 1 (*ESR1*), and synuclein alpha (*SNCA*). cDNA was synthesized from total RNA using a PrimeScript*™* RT reagent kit with gDNA Eraser (Takara Bio, Dalian, China). The analysis of these genes expression was done with SYBR Premix Ex Taq*™* II (Takara) using CFX96*™* real-time PCR detection system (Bio-Rad Laboratories, Hercules, CA, USA) in a 96-well optical plate. The PCR conditions were 95°C for 30 s, followed by 40 cycles of 5 s at 95°C and 30 s at 60°C. The expression value for each gene was normalized to the expression level of human glyceraldehyde-3-phosphate dehydrogenase (*GAPDH*), which was used as an internal control. The forward primer and reverse primer were designed (Sangon*™* Biotech, Shanghai, China) as shown in Table 2. The Ct values of 3 replicates for all examined genes and the internal control per sample were used to calculate 2^−ΔΔCt^ values.

### 2.6. Statistical Analysis

All calculations were performed by independent-samples *t*-test and one-way ANOVA analysis of variance using SPSS statistical software (SPSS 17.0; SPSS Inc., Chicago, IL, USA). Data were presented as means ± SEM, and statistical significance was defined as *p* < 0.05.

## 3. Results

### 3.1. Bioinformatics Analysis of RT-PCR Autophagy Array Results

**Figure 1:**
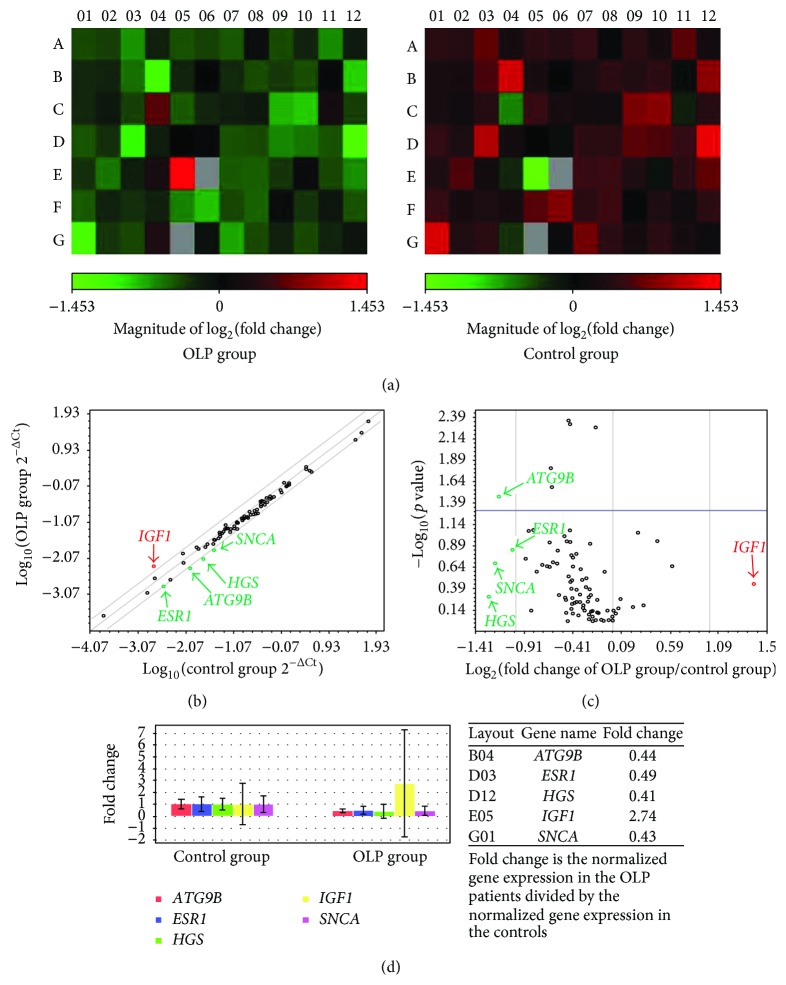
Different expression of autophagy-associated genes in T cells of OLP. (a) Heat plot displayed different expression of autophagy-associated genes in peripheral blood T cells from OLP patients and controls. (b) Scatter plot showed expression of autophagy-associated genes, with a fold difference of 2. (c) Volcano plot showed the significant difference in expression of autophagy-associated genes on T cells between OLP patients and control individuals. Fold change and fold regulation bigger than 2 were highlighted in red; fold change less than 0.5 and fold regulation less than −2 were highlighted in green. (d) Multianalysis displayed obvious overexpressed* IGF1* mRNA and decreased expression of* ATG9B*,* HGS*,* ESR1*, and* SNCA* in T cells of OLP.

**Figure 2:**
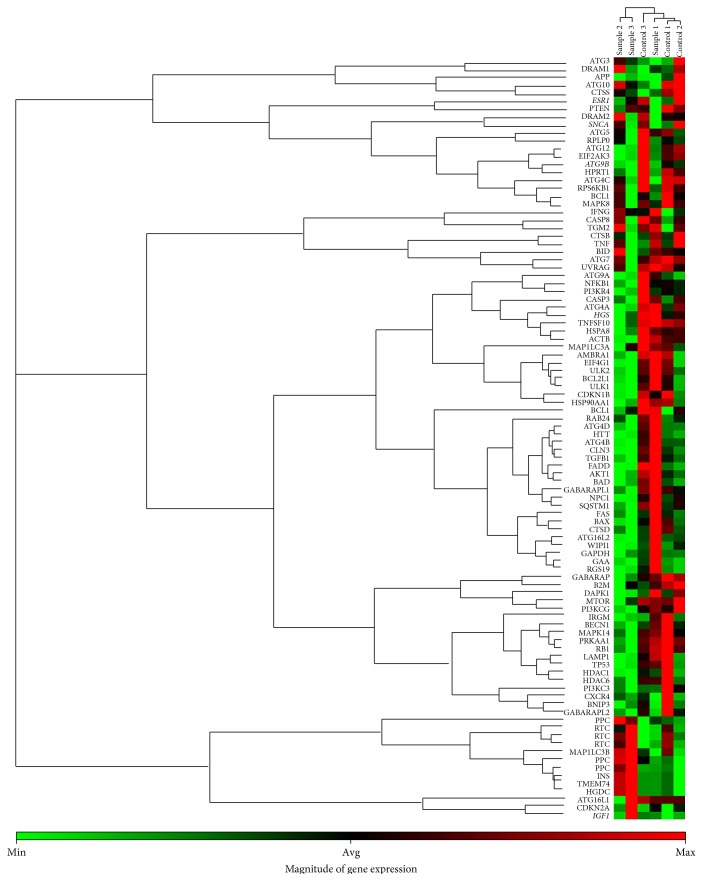
Expression patterns of autophagy-associated genes in OLP patients and controls based on autophagy array. Expression clusters were indicated by color bars next to the genes and subjects that were included in different clusters. The magnitude of gene expression was present at the bottom of the figure, which represented the gene mRNA relative expression levels by a range of color. Different autophagy-associated genes expression profiles in peripheral blood T cells were shown in two OLP forms and controls (sample 1: nonerosive OLP, NEOLP: sample 2 and sample 3: erosive OLP, EOLP, and control 1, control 2, and control 3: healthy controls).

The relative expression levels of the genes which met the criteria fold regulation > 2 or < −2 and fold change > 2 or < 0.5 were considered to be significantly different between OLP patients and controls. As shown in Figure 1, the expression of 84 autophagy-associated genes was measured in peripheral blood T cells of all subjects. Application of the criteria yielded a total of 5 differentially expressed genes. An increased expression of* IGF1* (fold regulation = 2.74; fold change = 2.74) and a decreased expression of* ATG9B* (fold regulation = −2.25; fold change = 0.44),* HGS* (fold regulation = −2.43; fold change = 0.41),* ESR1* (fold regulation = −2.04; fold change = 0.49), and* SNCA* (fold regulation = −2.31; fold change = 0.43) was found in the peripheral blood T cells of OLP patients. The results of hierarchical clustering analysis also indicated that these autophagy-associated genes were differentially expressed among patients with erosive and nonerosive OLP and controls (Figure 2).

### 3.2. Confirmation of the Differential Expression of* IGF1*,* ATG9B*,* HGS*,* ESR1*, and* SNCA* in T Cells between OLP Patients and Controls

**Figure 3:**
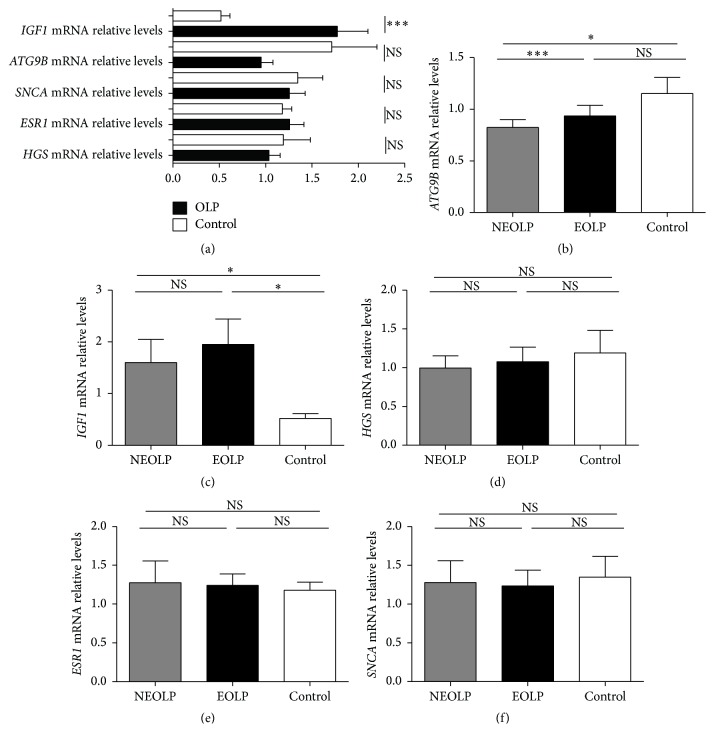
Expression of* IGF1*,* ATG9B*,* HGS*,* ESR1*, and* SNCA* of T cells in OLP patients and controls. (a) Expression of* IGF1*,* ATG9B*,* HGS*,* ESR1*, and* SNCA* mRNA in T cells of OLP patients (*n* = 22) and controls (*n* = 10). (b–f) The mRNA expression of* IGF1*,* ATG9B*,* HGS*,* ESR1*, and* SNCA* in nonerosive OLP patients (NEOLP, *n* = 11), erosive OLP patients (EOLP, *n* = 11), and healthy controls (*n* = 10). Data were shown as mean ± SEM (^*∗∗∗*^ *p* ≤ 0.001, ^*∗*^ *p* < 0.05, NS: nonsignificantly).

Next, confirmation of the autophagy array results was performed by quantitative real-time RT-PCR. The expression of* IGF1* mRNA was increased in the peripheral blood T cells of OLP patients, compared with that in the controls (OLP versus control: 1.78 ± 0.33 versus 0.52 ± 0.1, *p* = 0.001). However, there were no significant differences in the expression of* ATG9B*,* HGS*,* ESR1*, and* SNCA* between the OLP group (*n* = 22) and control group (*n* = 10) (Figure 3(a)).

### 3.3. Expression of* IGF1*,* ATG9B*,* HGS*,* ESR1*, and* SNCA* in T Cells from Patients with Different Clinical Forms of OLP

The results showed that* ATG9B* was differentially expressed between erosive and nonerosive OLP patients (Figure 3(b)). Overall,* ATG9B* mRNA expression was decreased in nonerosive OLP patients compared with that in controls and erosive OLP patients (nonerosive OLP versus control: 0.56 ± 0.09 versus 1.71 ± 0.49, *p* = 0.044; nonerosive OLP versus erosive OLP: 0.56 ± 0.09 versus 1.34 ± 0.18, *p* = 0.001). There were no significant differences in the expression levels of* IGF1*,* HGS*,* ESR1*, and* SNCA* between erosive (*n* = 11) and nonerosive OLP (*n* = 11) patients (Figures 3(c)–3(f)).

### 3.4. Differences in the Expression of* IGF1*,* ATG9B*,* HGS*,* ESR1*, and* SNCA* in T Cells between Male and Female OLP Patients

**Figure 4:**
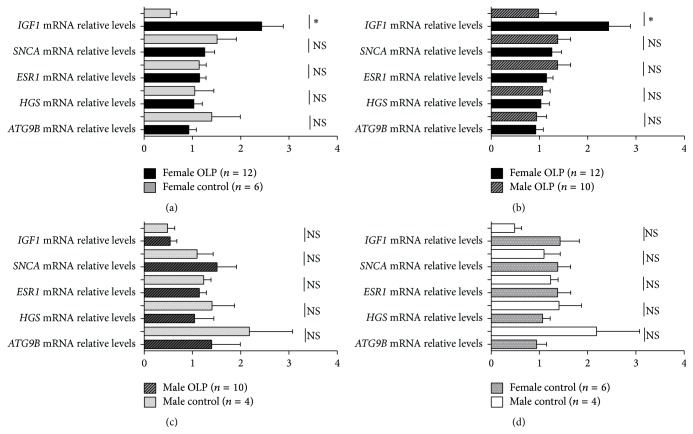
Expression of* IGF1*,* ATG9B*,* HGS*,* ESR1*, and* SNCA* mRNA in T cells from different genders of OLP patients and controls. Data were presented as mean ± SEM (^*∗*^ *p* < 0.05; NS: nonsignificantly).

Interestingly,* IGF1* mRNA expression in female OLP patients was obviously increased than that in female controls (OLP versus control: 2.43 ± 0.45 versus 0.64 ± 0.18, *p* = 0.042) (Figure 4(a)).* IGF1* mRNA expression in female OLP patients was also higher than that in male OLP patients (Female OLP versus Male OLP: 2.43 ± 0.45 versus 0.98 ± 0.36, *p* = 0.024) (Figure 4(b)). However,* ATG9B*,* HGS*,* ESR1,* and* SNCA* mRNA expression in T cells showed no significant differences between male (*n* = 10) and female OLP (*n* = 12) patients or between female OLP patients and female controls (Figures 4(b) and 4(a)). No significant differences in* IGF1*,* ATG9B*,* HGS*,* ESR1*, and* SNCA* mRNA expression were detected between male OLP patients and male controls or between male (*n* = 4) and female (*n* = 6) controls (Figures 4(c) and 4(d)).

### 3.5. Differences in the Expression of* IGF1*,* ATG9B*,* HGS*,* ESR1*, and* SNCA* in T Cells among OLP Patients of Different Ages

**Figure 5:**
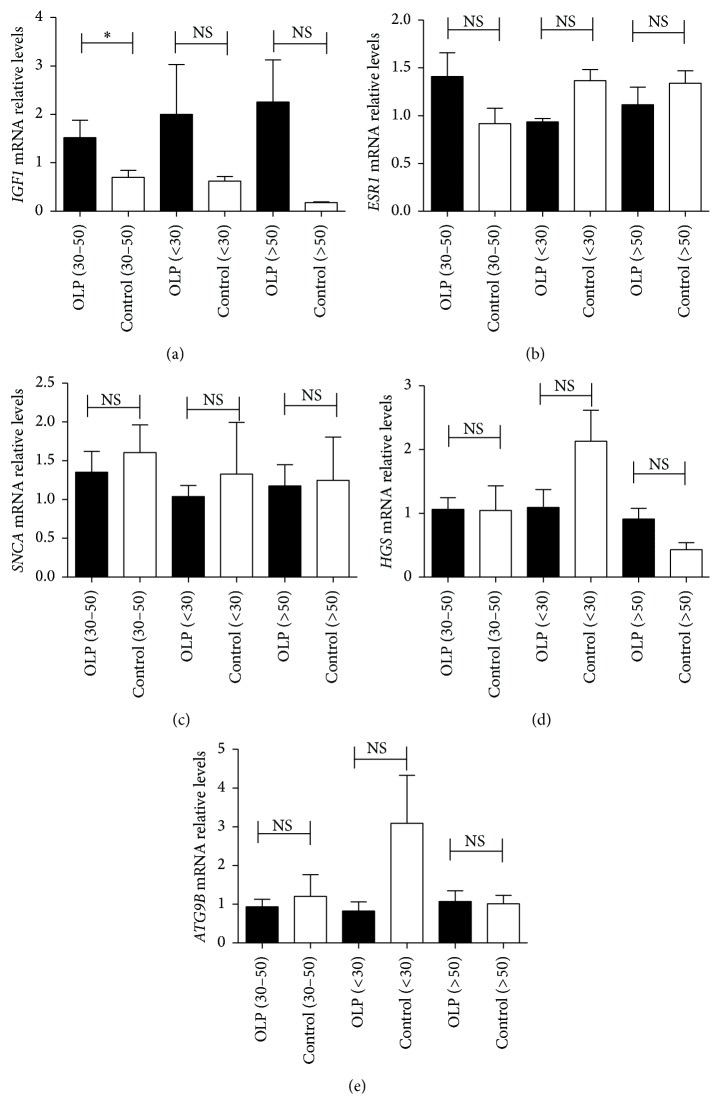
Expression of* IGF1*,* ATG9B*,* HGS*,* ESR1*, and* SNCA* mRNA in T cells from different age groups of OLP patients. The subjects were divided into three age groups, including those aged < 30 (OLP patients [*n* = 4] versus controls [*n* = 3]), those aged 30–50 years old (OLP patients [*n* = 13] versus controls [*n* = 4]), and those aged > 50 (OLP patients [*n* = 5] versus controls [*n* = 3]). Data were shown as mean ± SEM (^*∗*^ *p* < 0.05, NS: nonsignificantly).

Notably,* IGF1* mRNA expression was elevated in middle-aged (30–50 years old) OLP patients (OLP (*n* = 13) versus control (*n* = 4): 1.63 ± 0.37 versus 0.7 ± 0.15, *p* = 0.035). However,* IGF1* mRNA expression was not significantly different between OLP patients aged < 30 years (*n* = 4) and > 50 years (*n* = 5) (Figure 5(a)). No significant differences were found in* ATG9B*,* HGS*,* ESR1*, and* SNCA* mRNA expression between OLP patients and controls in all age groups (Figures 5(b)–5(e)).

## 4. Discussion

Accumulating evidence has shown that autophagy-associated genes may regulate immune signaling in a cell type specific way [15], but the role of autophagy in the T cells of OLP patients is still unknown. The analysis of autophagy-associated genes expression using RT-PCR arrays can provide insights into the autophagic function in the cells of patients, which can help to generate new hypotheses concerning the pathogenesis of complex disorders [23]. The array screening approach greatly shortens the time needed to analyze gene expression in complex biological systems, which may lead to the discovery of potential biomarkers for disease maintenance or offer clues for the development of novel therapies. The results of the autophagy-associated gene expression array used in this study demonstrated an altered expression of autophagy-associated genes in the T cells of OLP patients.

The present study showed that* IGF1* expression was upregulated in the peripheral blood T cells of OLP patients. IGF1 signaling could reduce cell death and control the potential exacerbation of the autophagic response under conditions of nutritional stress [24–26]. As an important regulator of autophagy, IGF1 can modulate the immune functions of peripheral lymphocytes [27]. Previous studies found that a chronic elevation of* IGF1* expression exacerbated mouse experimental autoimmune encephalomyelitis (EVE), which might be partly mediated by the expansion of T cells [27]. Moreover, IGF1 and its signaling axis have the potential to enhance myelin-specific T cell responses and impact the differentiation of CD4^+^ T cells into subsets such as Th1 and Th17 cells in EVE [27, 28]. Increased* IGF1* expression might also regulate the activation of CD8^+^ T cells by inducing p53 gene hypermethylation and could contribute to the polarization of antigen-specific CD8^+^ T cells in asthma [29]. Our previous studies implicated an imbalance between the Th1/Th2 immune response in OLP and showed that these responses had a predominant Th1 bias [4, 6, 7]. We detected the expression of two Th1/Th2-specific transcription factors,* T-bet* and* GATA-3*, in peripheral blood mononuclear cells and found that the expression of* T-bet* and the ratio of* T-bet*/*GATA-3* mRNA in OLP subjects were significantly higher than those in controls [7]. We also obtained results suggesting that OLP may be characterized by elevations of Th1 chemokines and cytokines, including C-C chemokine receptor 5 (CCR5), interleukin- (IL-) 2, and interferon- (IFN-) *γ* [4, 6]. In addition, Th17 cells and Th17-associated cytokines may be involved in the immune regulation of OLP [8]. Based on these findings, we speculated that the increased expression of* IGF1* in T cells from patients with OLP may mediate the immune response in OLP by regulating the T cell proliferation, differentiation, and activation.

Interestingly, the present data indicated a higher* IGF1* mRNA expression in the peripheral blood T cells of female and middle-aged OLP patients, compared with males and other age groups, respectively. Circulating IGFs are important regulators of prenatal and postnatal growth, and their abundances vary with genders and ages [30].* IGF1* was also found to be differentially expressed in condylar cartilage from a rat model of malocclusion among different genders and age groups [31]. Previous studies, including ours, have identified variations in the immunoreactivity of OLP among different genders and age groups [7]. OLP affects women more frequently than men, at a ratio of approximately 1.4 : 1 and occurs predominantly in middle-aged adults [3]. Taken together, we conjectured that* IGF1* expression in T cells may contribute to OLP in a gender-dependent and age-associated manner.

ATG9 is the integral multispan transmembrane protein among the core ATG proteins, which are required for autophagosome formation [32, 33]. The functions of ATG9 include the regulation of autophagy and the inhibition of innate immune signaling [12]. T cell autophagy appears to be abnormally regulated in autoimmune diseases such as multiple sclerosis and systemic lupus erythematosus [34, 35]. The array analysis performed in the present study showed that* ATG9B* expression in T cells was lower in OLP patients than in controls. A reduced expression of* ATG9B*, to some extent, might lead to autophagy dysregulation in the T cells of OLP patients. Notably, the mRNA expression of* ATG9B* in T cells was significantly decreased in patients with nonerosive OLP, when compared with that in controls and patients with erosive OLP. ATG9B, also known as endothelial nitric-oxide synthase antisense, is robustly induced by hypoxia [36]. Under hypoxic conditions, oral mucosa epithelial cells mainly undergo apoptosis in the lesion area of OLP [37]. The expression clusters of autophagy-associated genes in OLP suggested that differences in T cell autophagic activity may be associated with the different clinical presentation. Besides mediating the survival and homeostasis of cells, autophagy has been implicated both in antigen processing and presentation and in the secretion of proinflammatory cytokines such as type I IFN and tumor necrosis factor- (TNF-) *α* [12]. This latter function of autophagy is especially important in OLP, given that T cells regulate the apoptosis of basal keratinocytes via the secretion of TNF-*α*, especially in the more advanced stages of OLP [38]. Erosive OLP, which is featured by more evident oral mucosal damage, is generally considered to be a more severe form than nonerosive OLP [39]. Thus, we speculated that the decreased expression of* ATG9B* in T cells of nonerosive OLP patients might represent compromised autophagy, which could lead to less apoptosis of keratinocytes. In conclusion,* ATG9B* in T cells of OLP may contribute to the epithelial damage in different clinical forms.

The downregulation of other autophagy-associated genes including* HGS*,* ESR1*, and* SNCA* in peripheral blood T cells of OLP patients was indicated by the array analysis.* HGS* encodes a protein that regulates endosomal sorting and plays a critical role in the lysosomal transport, recycling, and degradation of ubiquitinated membrane proteins in the multivesicular body, which may be a key machinery for autophagic regulation [40].* ESR1* is essential for sexual development and reproductive function and is involved in the induction of autophagy in response to toxins [41, 42]. Autophagy inhibition can reduce intracellular SNCA aggregation and enhance SNCA secretion, which leads to an exacerbation of the microenvironmental response, including inflammation and cellular damage [43]. However, the confirmatory experiments using quantitative real-time RT-PCR showed no significant differences in the expression of* HGS*,* ESR1*, and* SNCA* in the peripheral blood T cells of OLP patients. These results suggested that a larger cohort of patients might yield further insights into autophagy-associated gene expression in OLP.

## 5. Conclusion

In summary, this study is the first to have analyzed the mRNA expression of autophagy-associated genes in peripheral T cells of OLP patients using autophagy array technology. Our results revealed an upregulation of* IGF1* expression on T cells in a gender-dependent and age-associated manner and a distinct expression pattern of* ATG9B* between different clinical forms, which may suggest the role of autophagy in the immune response of OLP. Further studies concerning the mechanisms of IGF1 and ATG9B in T cells autophagy of OLP are needed and may provide new therapeutic strategies for OLP.

## Figures and Tables

**Table 1:** Clinical features of the subjects.

	OLP (*n* = 25)	Control (*n* = 13)
Gender		
Male	12	5
Female	13	8
Age (years)		
Range	19~57	20~55
Mean ± SD	40.2 ± 2.4	34.5 ± 3.3
Clinical form		—
Nonerosive	12	
Erosive	13	

**Table 2:** Primer pairs designed for quantitative real-time RT-PCR analysis.

Gene	GenBank accession number	Primer sequences	PCR product size (bp)
*IGF1*	NM_000618	Forward: 5′-TCCTCGCATCTCTTCTACCTG-3′	153
		Reverse: 5′-ATACCCTGTGGGCTTGTTGA-3′	

*ATG9B*	NM_173681	Forward: 5′-CCTCGTGCCCTGGAGATTA-3′	196
		Reverse: 5′-AGAACCGCATCAAAGAAAGC-3′	

*HGS*	NM_004712	Forward: 5′-AGTCTGAGGAGAGCCACGAG-3′	114
		Reverse: 5′-CCGAGTCATTGGTGATGCT-3′	

*ESR1*	NM_000125	Forward: 5′-GGCTACATCATCTCGGTTCC-3′	118
		Reverse: 5′-AGACTTCAGGGTGCTGGACA-3′	

*SNCA*	NM_000345	Forward: 5′-GGGCAAGAATGAAGAAGGAG-3′	150
		Reverse: 5′-CAAGAAACTGGGAGCAAAGA-3′	

*GAPDH*	NM_001289746	Forward: 5′-CTTTGGTATCGTGGAAGGACTC-3′	134
		Reverse: 5′-CAGTAGAGGCAGGGATGATGTT-3′	
